# Human Serum Albumin Based Nanodrug Delivery Systems: Recent Advances and Future Perspective

**DOI:** 10.3390/polym15163354

**Published:** 2023-08-10

**Authors:** Changyong Li, Dagui Zhang, Yujing Pan, Biaoqi Chen

**Affiliations:** 1 College of Chemical Engineering, Huaqiao University, Xiamen 361021, China; changyong.li@hqu.edu.cn; 2 Fujian Provincial Key Laboratory of Biochemical Technology & Institute of Biomaterials and Tissue Engineering, Huaqiao University, Xiamen 361021, China

**Keywords:** human serum albumin, drug delivery, biomedical applications, binding sites

## Abstract

With the success of several clinical trials of products based on human serum albumin (HSA) and the rapid development of nanotechnology, HSA-based nanodrug delivery systems (HBNDSs) have received extensive attention in the field of nanomedicine. However, there is still a lack of comprehensive reviews exploring the broader scope of HBNDSs in biomedical applications beyond cancer therapy. To address this gap, this review takes a systematic approach. Firstly, it focuses on the crystal structure and the potential binding sites of HSA. Additionally, it provides a comprehensive summary of recent progresses in the field of HBNDSs for various biomedical applications over the past five years, categorized according to the type of therapeutic drugs loaded onto HSA. These categories include small-molecule drugs, inorganic materials and bioactive ingredients. Finally, the review summarizes the characteristics and current application status of HBNDSs in drug delivery, and also discusses the challenges that need to be addressed for the clinical transformation of HSA formulations and offers future perspectives in this field.

## 1. Introduction

Human serum albumin (HSA) is the most abundant protein in human plasma, with concentrations ranging from 35–50 g/L, accounting for approximately 50% of the total plasma protein [1]. Serving as a natural transporter in the bloodstream, HSA exhibits reversible binding capabilities with a range of endogenous and exogenous substances, such as fatty acids, hormones and metal ions [2]. It efficiently transports these substances to their targeted tissues. A variety of albumin-binding proteins (ABPs), including neonatal Fc receptor (FcRn), glycoprotein 60 kDa (gp60), and secreted protein acidic and rich in cysteine (SPARC), have been studied extensively [3]. They have also been demonstrated to be essential in the regulation of HSA circulation and distribution in the body. The intrinsic properties of HSA make it an attractive drug vehicle for delivering a variety of therapeutic agents. Over the past few decades, the extensive development of nanotechnology has also facilitated the development of drug delivery systems [4]. HSA, owing to its superior biodegradability and biocompatibility, non-toxicity, and non-immunogenicity, has been widely explored as a multifunctional nanodrug delivery system for biomedical applications. Various drugs can be combined to HSA through covalent conjugation, electrostatic adsorption, or hydrophobic interaction. As a result, HSA nanoparticles of different particle sizes can be easily fabricated using mild and facile strategies [5,6]. Furthermore, the presence of charged functional groups, including carboxyl and amino groups, provides possibilities for diverse surface modifications of HSA-based nanoparticles [7].

**Figure 1:**
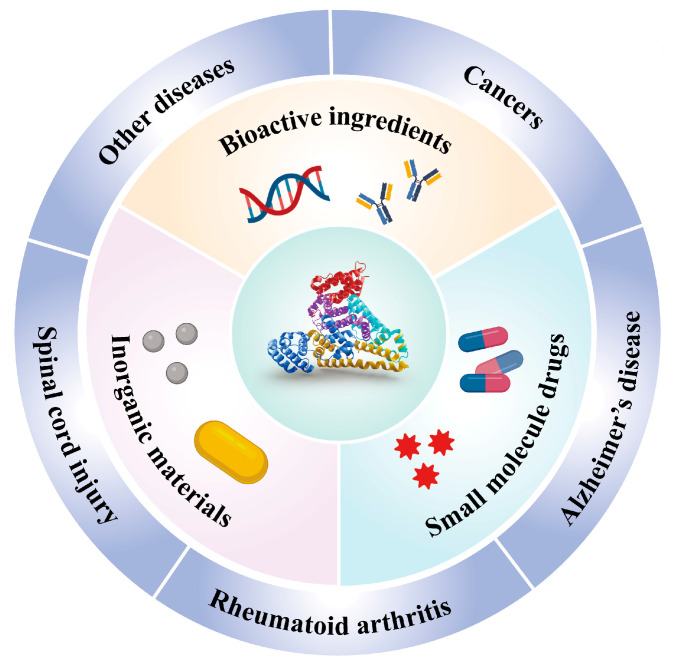
Overview of HSA-based nanocarriers for the delivery of diverse therapeutic agents (including small-molecule drugs, inorganic materials, and bioactive ingredients), and the potential biomedical applications of HSA-based nanocarriers.

Several HSA-based drug formulations have been approved for clinical trials and marketed. Among them is Abraxane, the first HSA-based product to be approved by the Food and Drug Administration (FDA) for human use in 2005 [8]. This formulation is manufactured by Nab™ technology and is clinically utilized in the treatment of breast cancer, non-small cell lung cancer, and pancreatic cancer. Furthermore, numerous other products are currently undergoing pre-clinical and clinical trials for a variety of biomedical applications, including cancers, diabetes, hemophilia, and rheumatoid arthritis (RA) [9]. These aforementioned facts have increased researchers’ interest in employing HSA as a nanocarrier for various biomedical applications. However, up until now, there has been a lack of specialized reviews specifically focusing on HSA-based nanocarrier for biomedical applications. In this review, our primary focus is on the application of HSA-based nanodrug delivery systems (HBNDSs) in the biomedical field (Figure 1), with particular emphasis on the recent progress made within the past five years. We hope that this review will provide a comprehensive understanding of HBNDSs, especially their applications in the biomedical field, and bring some possible inspiration to the clinical translation of HSA-based nanoformulations in the future.

## 2. Structure and Properties of HSA

HSA, synthesized by liver parenchymal cells, is composed of 585 amino acid residues and has a molecular weight of 66,500 Da [10]. HSA is characterized by a low content of aromatic amino acids, with only one tryptophan present, but it possesses a significant number of amino acids containing carboxyl and amino groups [11,12]. These amino acid residues contribute to the exceptional solubility and stability of HSA. Notably, HSA demonstrates remarkable stability over a broad pH range (pH 4–9) and exhibits excellent tolerance to specific organic solvents, withstanding concentrations of up to 40% ethanol. Additionally, HSA can endure high temperatures without denaturation, maintaining its structural integrity for a duration of 10 h at 60 °C [13,14]. These exceptional properties enable HSA to retain its structural characteristics even under harsh processing conditions.

**Figure 2:**
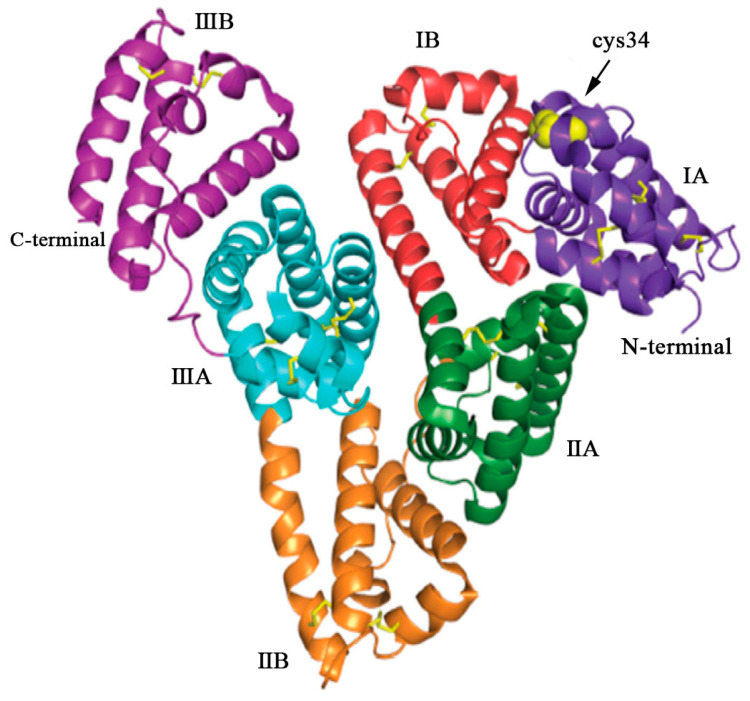
The structure of HSA, a single polypeptide consisting of 585 amino acids. HSA adopts a heart-shaped configuration, assembled by three homologous domains: domain I (residues 1–197), domain II (residues 189–385), and domain III (residues 381–585). Each domain comprises two subdomains, denoted as A and B, which exhibit common structural motifs. The domains are color coded: IA (purple), IB (red), IIA (green), IIB (orange), IIIA (blue), and IIIB (violet). The yellow sticks represent the disulfide bridges, while the yellow spheres represent the free cysteine residue located at position 34 (cys34) in domain IA. Reproduced with permission from the work of Kudarha et al. (2017) [18].

Figure 2 depicts the crystal structure of HSA, highlighting the specific binding sites for therapeutic drugs. In terms of structure, HSA consists of three homologous domains (domain I, II, and III), each of which is further divided into two subdomains, A and B. Each subdomain consists of six helical structures, and within each subdomain, hydrophobic and positively charged groups form a pocket-like structure. This arrangement creates an advantageous spatial environment for encapsulating hydrophobic nutrients, vitamins, hormones, and other molecules. As a result, HSA serves as a natural carrier for hydrophobic substances within the body. The unique spatial structural advantages of HSA facilitate its binding with a wide range of substances [15,16]. HSA possesses two major binding sites: Sudlow site I, located in subdomain IIA, and Sudlow site II, situated in subdomain IIIA (also known as the warfarin site and the benzodiazepine site). These sites exhibit high affinity for various molecules, including small-molecule drugs, peptides, nucleic acids, and so on [17]. Furthermore, HSA also has four metal binding sites, namely N-terminal binding site (NTS), cysteine 34 (cys34), and metal binding sites-A (MBS-A) and MBS-B, enabling the binding of different kinds of metal ions.

## 3. HSA-Based Multifunctional Nanocarrier

Due to its favorable attributes such as excellent biocompatibility, non-toxicity, non-immunogenicity, and prolonged circulation time, HBNDSs have garnered significant attention for a wide range of biomedical applications [11]. They have emerged as crucial carriers for delivering diverse therapeutic drugs, including small-molecule drugs, inorganic materials, and bioactive ingredients, thereby enhancing both imaging performance and therapeutic efficacy across various diseases [19]. In this section, we will systematically summarize the recent advancements in HSA-based multifunctional nanocarriers within the past five years.

### 3.1. Small-Molecule Drugs

The unique advantages of delivering small-molecule drugs using HSA as a carrier are as follows. (1) Good biocompatibility: HSA, being an endogenous substance, does not typically induce autoimmune reactions and adverse effects when used as a carrier. (2) High drug-loading ability. The unique spatial structure and abundant surface functional groups of HSA provide a convenient route for encapsulating diverse hydrophilic and hydrophobic small-molecule drugs in a high-affinity manner. (3) Prolonged half-life: When combined with drugs, HSA significantly extends the half-life time of drugs during the blood circulation. HSA itself exhibits a circulation half-life of approximately 19 days in the body. Additionally, HSA is negatively charged in the blood, making it less susceptible to clearance by macrophages. This feature provides the possibility of achieving prolonged blood circulation of drugs. (4) High stability: HSA can be utilized as a valuable carrier for exogenous drugs to improve their stabilities during systemic circulation and protect them from enzymatic degradation [20], thereby reducing drug leakage. The binding strategy between HSA and drugs is generally classified into two categories: covalent binding and non-covalent binding, based on the different approaches used to establish the interaction between HSA and the drugs. Typical examples of HSA-based nanocarriers for the delivery of small-molecule drugs within the past five years are summarized in Table 1.

#### 3.1.1. Covalent Binding

**Figure 3:**
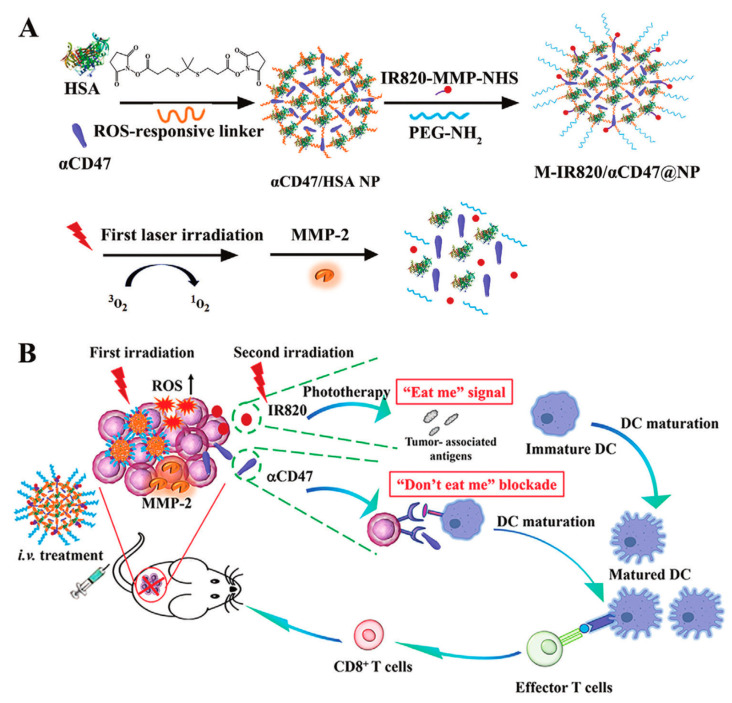
(**A**) Preparation and (**B**) simplified mechanism of M-IR820/αCD47@NP combining immunogenic cell death induction and CD47 blockade to improve cancer immunotherapy. Reproduced with permission from Lu et al. (2022) [22].

Theoretically, each HSA molecule contains one N-terminal carboxyl group, one cysteine (thiol group), and fifty-nine lysine residues (free amino group) [18]. Covalent binding refers to the preparation of HSA-drug nanoconjugate, by linking these abundant groups on the HSA with drugs through various chemical addition reactions, such as thiol-maleimide coupling, carbodiimide coupling, and Michael addition reactions [35]. In general, small-molecule drugs that can be combined with HSA through covalent binding include certain chemotherapeutic drugs, photosensitizers, and sonosensitizers. Examples of such drugs include methotrexate (MTX), doxorubicin (DOX), chlorine e6 (Ce6), and some prodrugs [36]. The nanoconjugates generated using this approach exhibit relatively high stability, reducing the potential side effects arising from drug leakage during blood circulation [37,38,39]. However, achieving controlled release of covalently connected drugs upon reaching the target sites has become a significant research focus in recent years. For instance, tumor tissue microenvironments possess distinctive characteristics, such as slight acidity and enzyme overexpression, in comparison to normal tissue [40]. A pH-sensitive linker, azidomethyl-methylmaleic anhydride (AzMMMan), was first employed to modify catalase (CAT) and HSA-Pt (IV) prodrug conjugates, respectively. Subsequently, a protein–drug conjugate (HSAP-DC-CAT, where DC represents dibenzocyclooctyne/chlorin) was synthesized by linking the two components through the click chemistry reaction between azide groups of AzMMMan and dibenzocyclooctyne (DBCO) groups of DBCO coupled Ce6 (DBCO-Ce6) [21]. The formation of stable covalent bonds between the drugs and HSA contributes to the systemic delivery of the nanoparticle, subsequently triggering trace lysis and release of bioactive Pt (IV), Ce6 and CAT in the acidic tumor microenvironment (TME). In addition to utilizing the special acidic TME, controlled drug release can also be achieved by establishing specific associations with overexpressed enzymes at the target site. In one instance, a light-activated, reactive oxygen species (ROS)-responsive nanoplatform (M-IR820/αCD47@NP) was designed by Lu et al. [22]. Notably, the photosensitizer IR820 was covalently linked by using a matrix metalloproteinase (MMP)-sensitive peptide as a linker and an αCD47@HSA NP with ROS responsiveness (Figure 3A). The M-IR820/αCD47@NP system releases the conjugated IR820 at the tumor site through MMP activation, enabling photodynamic therapy and inducing immunogenic cell death upon near-infrared (NIR) light irradiation. Additionally, the combination of CD47 blockade and photo-immunotherapy mediated by M-IR820/αCD47@NP elicits a significant antitumor immune response to effectively suppress the growth of 4T1 tumors and avoid tumor recurrence (Figure 3B).

Furthermore, the rapid growth and high metabolic activity of tumor tissues leads to inadequate blood supply, resulting in a hypoxic environment surrounding the tumor [41,42]. Exploiting the contrast between the low oxygen levels in tumor tissues and the normoxic conditions in normal tissues, it is possible to design hypoxia-sensitive nanoparticles. Although large nanoparticles (~100 nm) demonstrate excellent serum stability and accumulate in tumor tissues, their limited ability to penetrate deep-seated tumor regions restricts their efficacy. Conversely, small-sized nanoparticles (<20 nm) greatly enhance tissue penetration but exhibit reduced tumor accumulation [43]. This size contradiction hinders nanoparticles from achieving both effective tumor accumulation and deep tissue penetration. To overcome this limitation, Yang et al. developed a hypoxia-sensitive dissociable HSA nanoparticle [23]. They utilized the properties that azobenzene derivatives can be reduced to aniline derivatives under hypoxic conditions and various reductases [44,45]. By crosslinking the azobenzene group between HSA conjugated with the photosensitizer Ce6 (HC) and HSA conjugated with the oxaliplatin prodrug (HO), a size-tunable nanoplatform known as an HSA-based nanosystem (HCHOA) was created. This nanoplatform demonstrated favorable stability in the bloodstream, with an approximate diameter of 130 nm. Leveraging the enhanced permeability and retention (EPR) effect, the nanosized particle exhibited effective targeting of tumor tissues. Upon reaching the tumor site, the specific hypoxic environment triggered the cleavage of the azobenzene groups within the nanoparticles, leading to the disintegration of HCHOA into smaller HC and HO nanoparticles with sizes below 10 nm. This facilitated deep tissue penetration. Before and after dissociation, the photodynamic activity of Ce6 within HC experienced a transition from quenching to activation, resulting in an increase in singlet oxygen production. Ultimately, the combination of photodynamic therapy (PDT) and chemotherapy achieved highly efficient treatment of breast tumors.

#### 3.1.2. Non-Covalent Binding

As mentioned above, the crystal structure of HSA contains several specific binding sites that have the ability to bind various molecules with high affinity. One approach to non-covalent binding involves utilizing these inherent binding sites to form nanosized drug-HSA complexes. The available binding sites mainly include Sudlow sites I, Sudlow sites II, and cys34. Through reversible binding between HSA and drugs, the drug-HSA complexes can improve drug pharmacokinetics and enhance therapeutic efficacy by facilitating targeted accumulation and adequate drug release [46].

Another method of production of drug-loaded HSA nanoparticles involves encapsulating small-molecule drugs into the interior of nanoparticles during the process of HSA nanocrystallization; this can be achieved through techniques such as desolvation, emulsification method and self-assembly method [14]. The binding between drugs and HSA in this approach relies on hydrogen bonding interaction, hydrophobic interaction, and electrostatic adsorption. Compared with the covalent binding method, the non-covalent binding method is widely applicable and suitable for combining small-molecule drugs [8]. A smart nanoplatform was fabricated using a desolvation approach [29]. This involved integrating IR780 iodide (IR780) and piceatannol (PIC) into the core of the HSA nanoparticle, resulting in the formation of HSA/IR780/PIC NPs. These nanoparticles (NPs)were designed for photoacoustic imaging-guided combined cancer therapy, specifically enhanced sonodynamic therapy, and chemotherapy. In order to improve the therapeutic efficacy of HSA-bound drugs, it is crucial to understand how nanoformulation influences their behavior in TME. In another report by Li et al., a potential therapeutic strategy was proposed to enhance the effectiveness of nanoparticulate HSA-bound drugs in cancer treatment by reprogramming nutrient signaling and enhancing macropinocytosis in cancer cells [47]. The researchers revealed that the level of macropinocytosis in cancer cells can be upregulated in the manner of nutrient deprivation, which increases the accumulation of HSA-bound drugs in tumors and enhances the therapeutic efficacy.

Furthermore, the abundant functional groups present on the HSA allowed for the surface modification of active ligands, enhancing the targeting ability of the drug-loaded nanoparticles to the desired site. Arg-Gly-Asp (RGD) peptide, MMP-2 reaction sequence, and polyhistidine (pHis) were introduced at the end of the HSA by gene fusion technology, and PTX was loaded into the pHis micelle core through hydrophobic interaction [32]. Drug-loaded HSA nanoparticles with a three-stage propulsive effect (namely 3RGD-HSA-MMP-18His nanoparticles, RHMH18 NPs) were successfully constructed. This system aimed to achieve active tumor targeting, MMP-2 digestion-mediated deep penetration, and pH-responsive drug release. In vitro and in vivo experimental results demonstrated that RHMH18 NPs exhibited superior tumor growth inhibition and lower toxic side effects compared to Abraxane.

Another approach to enhance targeting is the utilization of microenvironment-sensitive HBNDSs, which enhance the responsiveness of nanoparticles to disease microenvironments such as pH, glutathione (GSH), and enzymes, allowing for controlled and sustained drug release. This strategy enables the nanoparticles to maintain their structural integrity under normal physiological conditions while selectively releasing the drug at the site of the lesion, minimizing damage to normal tissues and reducing toxic side effects [48]. GSH, a bioreductant present in human cells, exhibits significantly higher concentrations within cells compared to body fluids and extracellular matrix, with tumor tissues showing GSH levels over four times higher than normal tissues [49]. This disparity can be exploited to achieve reduction-triggered drug release. For instance, Zhang et al. employed non-covalent binding to encapsulate a disulfide bond bridged paclitaxel-pentadecanoic acid conjugate (PTX-SS-C_10_-COOH) within HSA, resulting in the formation of oxidation reduction-responsive nanoparticles (HPTX NPs) [31]. In vitro experiments demonstrated the nanoparticles’ stability under physiological conditions, with only 13.2% cumulative release of PTX within 30 h. In contrast, the nanoparticles exhibited faster and more complete PTX release, reaching a cumulative release of 81.3%, in simulated tumor microenvironment conditions (10 mM GSH). Moreover, compared to the commercialized Abraxane, these nanoparticles demonstrated enhanced tumor growth inhibition and lower biotoxicity.

Compared with free drugs, albumin-binding prodrugs tends to be more effective in cancer therapy. However, no clear studies have clarified the tumor-targeting ability of these prodrugs. Um et al. verified the in vitro and in vivo targeting efficiency of three albumin-binding molecules [50]. They are albumin-binding peptide (PEP), palmitic acid (PA), and maleimide (MI), which are labeled with the NIR fluorescent dye cyanine 5.5 (Cy5.5), respectively. Among these three compounds, PA-Cy5.5 bound to albumin non-covalently and formed the most stable complex, due to its reversible and multivalent binding affinities. In addition, PA-Cy5.5 showed the longest half-life (395.3 ± 192.9 h·µg/mL) and the highest tumor-targeting efficiency after intravenous injection into the tumor-bearing mice compared to the other two molecules. This suggests that albumin-binding molecules with reversible and multivalent affinities to native albumin would greatly improve their in vivo pharmacokinetics and enhance tumor-targeting efficiency.

### 3.2. Inorganic Materials

**Figure 4:**
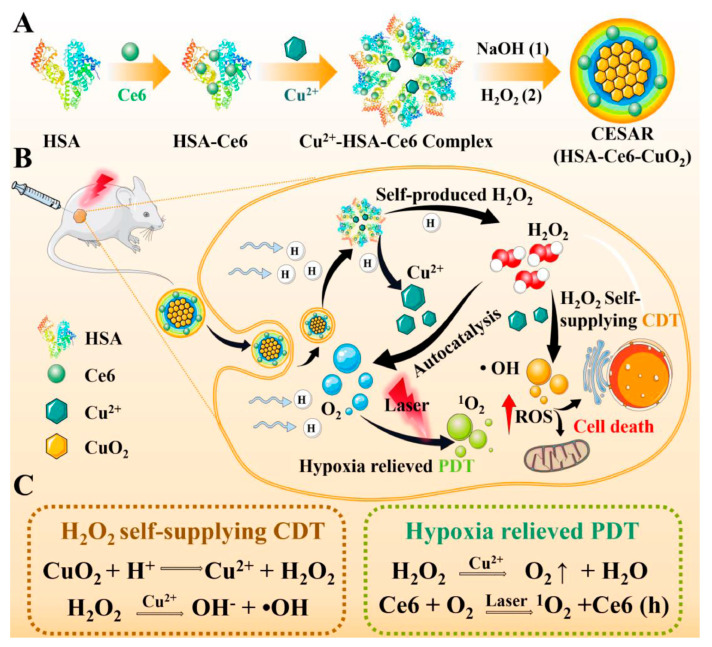
(**A**) The synthesis route of HSA-based tumor pH-responsive nanoreactor (CESAR). (**B**,**C**) The scheme of therapeutic mechanism of CESAR nanoreactor, which integrates the capacity of locally triggered H_2_O_2_/O_2_ self-supply and Cu^2+^ release for realizing efficient PDT/CDT. Reproduced with permission from Liu et al. (2022) [53].

In addition to small-molecule drug binding sites, the crystal structure of HSA also contains multiple metal binding sites. These binding sites allow for reversible binding of various metal ions (such as Ag^+^, Cu^2+^ and Mn^2+^), and they play an important role in the transport of metal ions during specific physiological or pathological processes in the living body [51]. The existence of these metal-binding sites has led to extensive exploration of HSA as a template for the synthesis of inorganic metal nanomaterials, including silver sulfide (Ag_2_S), gadolinium oxide (Gd_2_O_3_), manganese dioxide (MnO_2_), and copper sulfide (CuS) [8]. The process is similar to the biomineralization process occurring within the living organisms. Sun et al. constructed a NIR-II laser mediated photothermal Fenton nanocatalyst (PFN) by depositing MnO_2_ nanoparticles and CuS nanoparticles through a biomimetic biomineralization process using HSA as a stabilizer and template [52]. Due to the existence of CuS, PFN showed a good photothermal conversion efficiency under laser irradiation. This property not only enabled photothermal therapy (PTT), but also enhanced Cu^+^-mediated Fenton-like reaction. The combined effect of PFN-mediated PTT and chemodynamic therapy (CDT) exerted a synergistic ablation effect on xenograft tumors. However, the therapeutic efficacy of CDT is also critically dependent on the H_2_O_2_ level. Recently, a similar strategy was employed to develop a copper peroxide-based, tumor pH-responsive autocatalytic nanoreactor (CESAR), as reported by Liu et al. [53]. Upon exposure to the acidic TME, CESAR underwent collapse and instantly generated H_2_O_2_ and O_2_. The generated H_2_O_2_ facilitated the Cu^+^-catalyzed Fenton-like reaction, resulting in the production of a significant amount of ·OH for efficient CDT. Furthermore, the released O_2_ helped alleviate tumor hypoxia, thereby enhancing the efficacy of Ce6-mediated PDT (Figure 4). Overall, this strategy provides a promising paradigm to improve cancer therapies in cases of hyperoxide deficiency or oxygen limitation.

In addition to the mineralization of inorganic metal nanoparticles on the HSA, the surface functional groups of HSA can also be covalently linked to specific inorganic non-metallic nanoparticles. Alzheimer’s disease (AD) is the most prevalent form of dementia, characterized by the accumulation of amyloid-beta (Aβ) proteins and elevated levels of ROS [54,55]. Currently, AD affects over 50 million individuals worldwide [56], underscoring the urgent need for comprehensive diagnostic and therapeutic interventions. For instance, Wang et al. developed a multifunctional nanoparticle based on HSA (HSA-BFP) by introducing an Aβ fluorescent probe (F) and a cell-penetrating peptide (Penetratin, Pen) onto basified HSA (HSA-B) [57]. Further coupling of carbon dots (CDs) with HSA-BFP using N-succinimidyl-3-(2-pyridyldithio) propionate (SPDP) as a linker resulted in a targeted Aβ multifunctional protein-carbon dot conjugate (HSA-BFP@CDs). Upon interaction with Aβ aggregates, HSA-BFP@CDs exhibited a fluorescence signal at 700 nm, transitioning from an off state to an on state, showcasing the potential for early diagnosis of AD. Furthermore, in vitro and in vivo experiments utilizing Caenorhabditis elegans as a model demonstrated that HSA-BFP@CDs effectively suppressed Aβ aggregation and mitigated oxidative stress in vivo, underscoring the potential and prospects of protein-carbon dot conjugates in the multi-targeted treatment of AD.

### 3.3. Bioactive Ingredients

As a non-toxic, non-immunogenic, stable, and biocompatible material, HSA nanoparticles are also employed as useful vehicles for bioactive ingredients, including nucleic acids (DNA and RNA), antibodies, peptides, cytokines, and enzymes [58]. Recent studies of HSA-based nanoparticles for the delivery of bioactive ingredients and their biomedical applications are listed in Table 2. On the one hand, using HSA nanoparticles for the delivery of these bioactive ingredients can avoid unnecessary immunogenicity and potential safety issues. On the other hand, utilizing HSA nanoparticles as carriers can provide significant protection to these active components against enzymatic digestion, enhance their systemic stability during blood circulation, and effectively improve their pharmacokinetics.

**Figure 5:**
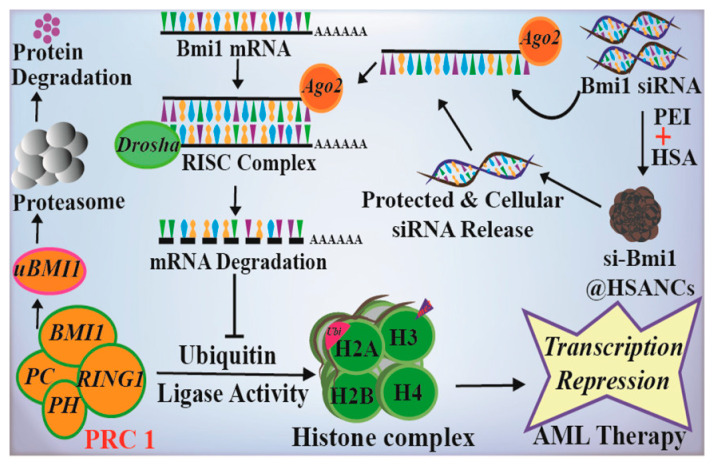
Schematic representation of the construction of HSA NPs-PEI@EZH2 siRNA and the possible mechanism of AML therapy. Reproduced with permission from the work of Kushwaha et al. (2020) [61].

AML is the most common hematological malignancy, with a high recurrence rate and poor long-term survival, accounting for 42% of all leukemia deaths [73]. Some studies have shown that the polycomb protein Bmi1 is overexpressed in AML and plays a role in disease development through its downstream targets [74], making it a potential epigenetic target. Kushwaha et al. proposed a delivery strategy using HSA nanoparticles as carriers (nanocarriers, NCs) for Bmi1 siRNA, which was stabilized by polyethylenimine (PEI) [61]. This innovative approach, known as PEI@HSANCs, aimed to enhance the effectiveness of Bmi1 siRNA delivery. The PEI-modified HSA nanocarrier demonstrated the ability to protect Bmi1 siRNA from degradation by ribonucleases, resulting in a significant increase in the transfection efficiency of Bmi1 siRNA through caveolae-mediated endocytosis (Figure 5). The siRNA transfection efficiency in the PEI@HSANCs group was nearly 2.4 times higher than that in the siRNA group. In another study, Kaundal et al. identified another member of the polycomb histone family, Enhancer of Zeste Homolog 2 (EZH2), which is highly expressed in AML [62]. The authors proposed an effective EZH2 siRNA delivery strategy based on HSA (HSANPs-PEI@EZH2siRNA). EZH2 siRNA was loaded in PEI-modified HSA nanoparticles, which displayed enhanced system stability and blood compatibility. These nanoparticles precisely targeted the EZH2 gene in AML cells, leading to enhanced EZH2 gene silencing.

RA is a prevalent chronic systemic autoimmune disease that poses a serious threat to human health and well-being [75]. In light of this, an HSA-based nanodrug co-loaded with the anti-RA medication MTX and the ROS scavenger superoxide dismutase (SOD) was designed, and is now known as HSA-SOD-MTX [71]. Owing to the overexpression of SPARC at arthritic inflammatory sites, HSA-SOD-MTX nanoparticles can specifically target these sites, thereby overcoming the challenges of insufficient affinity of SOD for the target and the lack of evident drug tropism. The SOD incorporated in the nanoparticles exhibited effective scavenging of O_2_^•−^ and notably reduced the expression levels of various in vitro inflammation-associated cytokines (granulocyte colony-stimulating factor G-CSF, monocyte chemotactic protein-1 MCP-1 as well as regulated on activation, normal T cell expressed and secreted RANTES), which is beneficial to the treatment of RA. In vivo results showed that the combined action of SOD-mediated ROS scavenging and MTX-mediated anti-rheumatism was able to significantly inhibit the inflammatory response in the joints of CIA mice without inducing obvious systemic toxicity.

## 4. Conclusions and Future Perspectives

Owing to its advantages of good biocompatibility, non-toxicity, non-immunogenicity, and long half-life, HSA has received much attention in the study of drug delivery systems. Utilizing the intrinsic binding sites of HSA and the extensive developments in nanotechnology for HSA nanocrystallization, therapeutic drugs can be effectively conjugated with HSA or encapsulated within HSA nanoparticles. These drugs include small-molecule drugs, inorganic materials, as well as bioactive ingredients (such as nucleic acids, peptides, antibodies, cytokines, and enzymes). They help to enhance systemic stability, improve pharmacokinetics, and enhance the therapeutic efficacy of drugs while minimizing systemic side effects. The existence of functional groups on HSA allows for their modification with multiple targeted ligands, enhancing the targeting of drug delivery systems and reducing the accumulation of therapeutic drugs in non-targeted regions. Additionally, HSA-based stimuli-responsive nanodrug delivery systems can be designed for on-demand drug release according to various covalent or non-covalent approaches, on the basis of the characteristics of the lesion microenvironment and the structural characteristics of HSA. Stimulus-sensitive HBNDSs can decompose and release drugs, under specific endogenous conditions (e.g., pH, GSH, and multiple enzymes) or exogenous conditions (e.g., laser and ultrasound), effectively increasing the drug concentration at the desired region. For example, due to the rapid growth and vigorous metabolism of tumor tissue, it requires a lot of energy and protein. Albumin serves as the main source of energy and amino acids for tumor tissue. A large number of receptors and proteins will be over-expressed on tumor cells, which can bind with HSA in a high-affinity manner. HSA-based nanoparticles can preferentially accumulate in tumors through this mechanism. Moreover, due to the increased albumin metabolism in tumors, the targeted delivery of cytotoxic agents to tumor tissue can be realized. This may have beneficial implications for the design of clinical antitumor drugs, such as targeted therapy for pan-KRAS mutant cancers [76]. Similarly, the overexpression of SPARC is also confirmed in arthritic inflammatory sites [77], compared to healthy tissues. These findings demonstrate the great potential of HBNDSs for use in the biomedical field.

Despite the unique advantages of this method, there are still some problems and mechanisms that need to be solved and explored. Firstly, in terms of cancer therapy, HBNDSs can passively target tumor tissues via the EPR effect, thus enhancing the efficacy of antitumor drugs. However, it remains to be validated whether similar nanoparticle enrichment mechanisms exist in other biomedical disease models. Secondly, it has been shown that the in vivo transport and distribution of HSA is regulated by ABPs. It is important to determine whether drug-loaded HSA nanoparticles are still recognized by cells and internalized through this pathway. Moreover, the in vivo transport mechanism of drug-loaded HSA nanoparticles also needs to be investigated. Further research on the structural changes, the distribution and transport of HSA nanoparticles after binding with drugs will play a key role in accelerating the development of HSA formulations. HSA also has its own defects as a drug carrier, such as the limited source and expensive cost. Recombinant HSA is a recently developed genetically engineered protein expressed by yeast cells and is promising as a potential candidate. The clinical challenge for HSA nanoformulations in future biomedical applications may be to look for raw materials with stable properties and highly reproducible preparation methods. Currently, research on HSA formulation-based approaches is ongoing. We expect that in the near future, more new HSA-based therapeutic and diagnostic products will be approved for clinical use, benefiting patients.

## Figures and Tables

**Table 1:** Typical examples of HSA NPs loaded with small-molecule drugs for various biomedical applications.

Material	Method	Size (nm)	Drug	Loading Capacity/ Encapsulation Efficiency (%)	Cell/Animal Model	Target Diseases	Ref.
HSAP-DC-CAT	Covalent binding	164	Pt (IV)SA; Ce6	-/74.2; -/73.2	4T1 cells/4T1 tumor-bearing mice	Breast cancer	[21]
M-IR820/αCD47@NP	Covalent binding	149.8 ± 11.0	IR820	80.2 ± 2.9 µg/mg/-	4T1 cells/4T1 tumor-bearing mice	Breast cancer	[22]
HCHOA	Covalent binding	100–150	Oxa(IV)-COOH; Ce6	1.82/-; 1.28/-	4T1 cells/4T1 tumor-bearing mice	Breast cancer	[23]
HRC@PTX	Covalent binding	174.1 ± 2.1	All-trans retinoic acid; Ce6	-	MCF-7 and MDA-MB-231 cells	Breast cancer	[24]
Nab-PTX-PA	Nab™ technology	87.6 ± 1.1	PTX-palmitate	18.30 ± 0.31/97.71 ± 0.49	4T1 cells/4T1 tumor-bearing mice	Breast cancer	[25]
N-PD/CU	High-pressure homogenization	150.4 ± 2.4	Prednisolone; Curcumin	-/88.75 ± 1.82; -/85.79 ± 1.43	RAW264.7 cells/Adjuvant- induced arthritis mice	Rheumatoid arthritis	[26]
dcHGT NPs	Desolvation	14.6	Clioquinol; Donepezil	-/41.0; -/35.0	BV2 cells/APP/PS1 AD mice	Alzheimer’s disease	[27]
Vitamin C–loaded HSA NPs	Desolvation	180 ± 6	Vitamin C	-/52.1	3T3 fibroblast cells	Wound healing	[28]
HIP NPs	Desolvation	152.6 ± 24.2	IR780 iodide; Piceatannol	1.94 ± 0.14/-; 6.39 ± 0.13/-	4T1 cells/4T1 tumor-bearing mice	Breast cancer	[29]
HSA-INH-RIF NPs	Desolvation	216.7 ± 3.7	Rifampicin (RIF); Isoniazid (INH)	44/-; 27/-	-	Tuberculosis	[30]
HPTX NPs	Desolvation and lyophilization	~120	PTX-SS-C10-COOH	29.78/94.16	MDA-MB-231 cells/MDA-MB-231 tumor-bearing mice	Breast cancer	[31]
RHMH18 NPs	Self-assembly	~100	PTX	6.59/-	A549 cells and MGC-803 cells/A549 and MGC-80 tumor-bearing mice	Lung cancer; Gastric cancer	[32]
HSA-RB-DOX NPs	Self-assembly	42.0	DOX; Rose bengal	8.5/-; 7.1/-	MCF-7 cells/MCF-7 tumor-bearing mice	Breast cancer	[33]
Pt(lau)HSA NPs/Rex	Nanoprecipitation	120–140	Laurate- functionalized Pt (IV) prodrug	-/23.15	4T1 cells/4T1 tumor-bearing mice	Breast cancer	[34]

**Table 2:** Typical examples of HSA-based nanocarriers for the delivery of bioactive ingredients and their biomedical applications.

Material	Size (nm)	Bioactive Ingredient	Function	Cell/Animal Model	Target Disease	Ref.
FeBcl2HD@PEI	~290	Bcl-2 siRNA	Silence mRNA of Bcl-2 gene; inhibit the expression of Bcl-2	A549 cells/-	Lung cancer	[59]
pDNA/DSP-NPs	242.5 ± 4.5	IL-10 pDNA	Express IL-10 efficiently; inhibit pro-inflammatory factors; facilitate macrophage polarization	RAW264.7 cells/Collagen-induced arthritis (CIA) mice	Rheumatoid arthritis	[60]
si-Bmi1@HSANCs	175.4 ± 40.4	Bmi1 siRNA	Degrade mRNA of Bmi1; downregulate the expression of Bmi1	U937 and HL60 cells/Acute myeloid leukemia (AML) xenograft mice	AML	[61]
HSANPs-PEI@EZH2siRNA	90.0 ± 22.2	EZH2 siRNA	Silence mRNA of EZH2 gene; inhibit the expression of EZH2	U937 and HL60 cells/AML xenograft mice	AML	[62]
INS (IL-12@HSA)	~50	IL-12	Recruit cytotoxic T cells; induce the production of antitumor cytokines	Raji cells/Raji tumor-xenografted mice	Lymph cancer	[63]
PD-L1/PTX@HSA	200–250	Anti-PD-L1 antibody	Enable tumor target and enhance antitumor efficacy	EMT-6 cells/EMT-6 tumor-xenografted mice	Breast cancer	[64]
M-IR820/αCD47@NP	149.8 ± 11.0	Anti-CD47 antibody	Block CD47 and activate antitumor immunity	4T1 cells/4T1 tumor-bearing mice	Breast cancer	[22]
HSA@NVax	70–80	PCSK9 peptide	Reduce circulating levels of LDL-C	DC2.4 and RAW264.7 cells/immunologically naive mice	Atherosclerotic cardiovascular disease	[65]
Cy7–B5–HSA–5-FU	208.2	B5 peptide	Enable active targeting	-/Colorectal cancer PDX mouse model	Colorectal cancer	[66]
R837-Pep@HM NPs	~120	Melanoma peptide	Activate tumor-specific immune responses	B16F10 cells/B16F10 melanoma tumor-bearing mice	Melanoma	[67]
AT1-HSA-MRN-NPs	215.2 ± 4.7	AT1 peptide	Enable active targeting	H9c2 cells/-	Congestive heart failure	[68]
TAT-TMP-NPs	-	HIV TAT peptide	Improve nanoparticles’ uptake and efficiency of delivery by neutrophils	BV2 and SH-SY5Y cells/Spinal cord injury (SCI) male mice model	SCI	[69]
^131^I-HSA-CAT NRs	~100	Catalase	Decompose H_2_O_2_ to generate oxygen and improve the therapeutic efficacy of radionuclide therapy	4T1 cells/4T1 tumor-bearing mice	Breast cancer	[70]
HSA-SOD-MTX	78.0 ± 12.4	SOD	Scavenge ROS and decrease the expression levels of pro-inflammatory cytokines	RAW264.7 cells/CIA mice	Rheumatoid arthritis	[71]
HO-HM/GOD	~140	Glucose oxidase	Catalyze glucose to produce H_2_O_2_ and enhance CDT efficacy	4T1 cells/4T1 tumor-bearing mice	Breast cancer	[72]

## Data Availability

Not applicable.

## Abbreviations

ABPs	Albumin binding proteins.
AD	Alzheimer’s disease.
Ag_2_S	Silver sulfide.
AML	Acute myeloid leukemia.
AT1	Angiotensin II type 1.
AzMMMan	Azidomethyl-methylmaleic anhydride.
Aβ	Amyloid-beta.
Bcl-2	B-cell lymphoma-2.
CAT	Catalase.
Ce6	Chlorine e6.
CIA	Collagen-induced arthritis.
CDs	Carbon dots.
CDT	Chemodynamic therapy.
CESAR	Copper peroxide-based tumor pH-responsive autocatalytic nanoreactor.
CU	Curcumin.
CuS	Copper sulfide.
Cy5.5	Cyanine 5.5.
cys34	Cysteine 34.
DBCO	Dibenzocyclooctyne.
DBCO-Ce6	DBCO coupled Ce6.
DC	Dibenzocyclooctyne/chlorin.
DOX	Doxorubicin.
DSP	Dexamethasone sodium phosphate.
EPR	Enhanced permeability and retention.
EZH2	Enhancer of Zeste Homolog 2.
FcRn	Fc receptor.
FDA	Food and Drug Administration.
G-CSF	Granulocyte colony-stimulating factor.
Gd_2_O_3_	Gadolinium oxide.
GOD	Glucose oxidase.
gp60	Glycoprotein 60 kDa.
GSH	Glutathione.
HSA	Human serum albumin.
HBNDSs	HSA-based nanodrug delivery systems.
HC	HSA conjugated with Ce6.
HO	HSA conjugated with oxaliplatin.
HCHOA	A nanoplatform by crosslinking azobenzene group between HC and HO.
HPTX	HSA conjugated with PTX-SS-C_10_-COOH.
HSA-BFP	A multifunctional nanoparticle based on basified HSA (HSA-B) by incorporating an Aβ fluorescent probe (F) and a cell-penetrating peptide (Penetratin, Pen).
HSAP	HSA-Pt (IV).
IL-10	Interleukin-10.
INH	Isoniazid.
IR780	IR780 iodide.
LDL-C	Low-density lipoprotein cholesterol.
MBS-A	Metal binding sites-A.
MBS-B	Metal binding sites-B.
MCP-1	Monocyte chemotactic protein-1.
MI	Maleimide.
MMP	Matrix metalloproteinase.
MnO_2_	Manganese dioxide.
MRN	Milrinone.
MTX	Methotrexate.
NCs	Nanocarriers.
NIR	Near-infrared.
NP(s)	Nanoparticle(s).
NTS	N-terminal binding site.
PA	Palmitic acid.
PCSK9	Proprotein convertase subtilisin/kexin type 9.
PD	Prednisolone.
PD-L1	Programmed cell death-ligand 1.
pDNA	Plasmid DNA.
PDT	Photodynamic therapy.
PDX	Patient-derived xenograft.
PEI	Polyethylenimine.
PEP	Albumin-binding peptide.
PFN	Photothermal Fenton nanocatalyst.
pHis	Polyhistidine.
PIC	Piceatannol.
PTT	Photothermal therapy.
Pt (IV)SA	Succinic acid-derivatized cisplatin prodrug.
PTX	Paclitaxel.
pVax	Peptide vaccine.
RA	Rheumatoid arthritis.
RB	Rose bengal.
RANTES	Regulated on activation, normal T cell expressed and secreted.
RGD	Arg-Gly-Asp.
RIF	Rifampicin.
ROS	Reactive oxygen species.
SCI	Spinal cord injury.
siRNA	Small interfering RNA.
SOD	Superoxide dismutase.
SPARC	Secreted protein acidic and rich in cysteine.
SPDP	N-succinimidyl-3-(2-pyridyldithio) propionate.
TAT	Trans-activator of transcription.
TME	Tumor microenvironment.
TMP	Tetramethylpyrazine.
5-FU	5-Fluorouracil.
